# Inverse relationship between serum vitamin D level and measles antibody titer: A cross-sectional analysis of NHANES, 2001-2004

**DOI:** 10.1371/journal.pone.0207798

**Published:** 2018-11-30

**Authors:** Yi-Hsien Chen, Wei-Ming Wang, Tung-Wei Kao, Chien-Ping Chiang, Chih-Tsung Hung, Wei-Liang Chen

**Affiliations:** 1 Department of Dermatology, Tri-Service General Hospital; and School of Medicine, National Defense Medical Center, Taipei, Taiwan, Republic of China; 2 Division of Family Medicine, Department of Family and Community Medicine, Tri-Service General Hospital; and School of Medicine, National Defense Medical Center, Taipei, Taiwan, Republic of China; 3 Division of Geriatric Medicine, Department of Family and Community Medicine, Tri-Service General Hospital; and School of Medicine, National Defense Medical Center, Taipei, Taiwan, Republic of China; 4 Graduate Institute of Clinical Medical, College of Medicine, National Taiwan University, Taipei, Taiwan, Republic of China; University of Auckland, NEW ZEALAND

## Abstract

**Background:**

In recent years, researchers have illuminated many non-skeletal actions of vitamin D including host defense against various pathogens and vaccine immunology. The purpose of our study was to explore the potential association between serum vitamin D levels and measles antibody titers.

**Methods:**

The biochemical profiles and de-identified information were accessed from the 2001 to 2004 National Health and Nutrition Examination Survey (NHANES). Participants were divided into quartiles according to their measles antibody titers.

**Results:**

A total of 5,681 participants were analyzed in our study. Participants in the highest quartile of measles antibody titer had significantly lower serological levels of 25-hydroxyvitamin D [25(OH)D] than those in the lower quartiles (53.90 vs. 58.70 nmol/L, a decrease of 8.18%) (p < 0.001). After full adjustment of confounders, the adjusted ß coefficient of 25(OH)D was -0.006 (p<0.001). A decreasing tendency of 25(OH)D among quartiles of measles antibody titers was obvious (p for trend <0.001). The negative association in seropositive subjects remained statistically significant only in non-Hispanic black population before adjustment for age, gender, and other covariates (p<0.05).

**Conclusion:**

Our study highlights the negative association between serum 25(OH)D levels and measles antibody titers.

## Introduction

Vitamin D, traditionally known to be involved in calcium phosphate homeostasis and mineral metabolism, has been proven to be a pleiotropic molecule.[1] Emerging evidence has linked the serum concentration of vitamin D to various constitutional conditions, including autoimmune diseases, cardiovascular diseases, and cancer.[2] Patients with lower vitamin D levels were found to have increased disease activity in multiple sclerosis and inflammatory bowel disease.[3] 1,25(OH)_2_D, an active form of vitamin D, was shown to augment the fusion of lysosomes and phagosomes in Mycobacterium tuberculosis infected macrophages.[4] Additionally, vitamin D has been reported to play a role in systemic and mucosal immune responses in animal studies.[5, 6]

Measles, or Rubeola, is a highly contagious disease and can cause fever, conjunctivitis, morbilliform rash, pneumonia, diarrhea, encephalitis, and death. A dramatic decline in the incidence of measles to less than one case per million has been achieved since 1997 following the increase of vaccination of school-age children.[7–9] In March 2000, the transmission of the measles virus was declared eliminated in the United States.[10] However, cases of measles continued to occur, mainly associated with emigration, because the disease is still endemic in many developing countries.

Researchers have been interested in the mechanism of how vitamin D affects human immunity against various infectious diseases and the immune response to vaccines. Vitamin D deficiency was found to be associated with poor response to hepatitis B immunization in patients with chronic kidney disease.[11] Furthermore, another study demonstrated that genetic variants in vitamin D receptors determine the inter-individual immune response to the measles vaccine.[12] However, vitamin D levels were not assessed in that study. To the best of our knowledge, the impact of vitamin D levels on the immune response to either a natural measles infection or to the measles vaccine has not been studied. Here, we focused on the potential connection between vitamin D and measles antibody titers.

## Materials and methods

### Ethics statement

The NHANES databank where we obtained our de-identified information was approved by the Institutional Review Board of the National Center for Health Statistics (NCHS IRB). All informed consent was obtained before data collection and health examinations. The entire experimental protocol was approved by the NCHS IRB.

### Data source and study population

We initially identified 15,206 participants with available standardized serum 25(OH)D data from NHANES 2001–2004.[13, 14] Participants who finished biochemical examination, serum 25(OH)D, measles antibody titer, and questionnaire of past histories were included in our study. Based on the inclusion criteria of our study, the final analytical sample was 5,681 participants showed in Table 1. Secondary data analysis was performed on serum vitamin D, measles antibody titer, and other covariates obtained from the NHANES 2001–2004. The NHANES are a series of nationwide consecutive cross-sectional surveys of the representative non-institutionalized US population since 1999. NHANES, conducted by NCHS of the Centers for Disease Control and Prevention (CDC), have a stratified multistage probability design. Minority groups of certain ethnicities, such as non-Hispanic black Americans and Mexican Americans, certain ages, and certain income levels were oversampled to make stable estimates for these groups. The NHANES also comprised in-home interviews and health examinations in a mobile examination center. Descriptions of detained operation instructions and documentation of informed consent for the NHANES 2001–2004 were available on the internet for public download.

**Table 1:** Characteristics of study participants.

	Quartiles of measles antibody titer					
Variables	Q1(<4.22)	Q2(4.22to<8.38)	Q3(8.38to<14.145)	Q4(≥14.145)	total	p value
	N = 1421	N = 1423	N = 1417	N = 1420	N = 5681	
Continuous variables ^a^						
Age (years)	29.00 (12.00)*	31.00 (14.00)*	36.00 (14.00)*	41.00 (12.00)	34.00 (16.00)	<0.001
Vitamin D (nmol/L)	58.70 (28.60)*	58.70 (31.00)*	56.30 (31.10)*	53.90 (30.90)	56.30 (31.00)	<0.001
ALT (U/L)	20.00 (14.00)	21.00 (14.00)	20.00 (14.00)	22.00 (14.00)	21.00 (14.00)	0.158
Total cholesterol (mg/dL)	190.00 (53.00)	194.00 (57.00)	194.00 (53.00)	193.00 (49.00)	193.00 (53.00)	0.564
Glucose (mg/dL)	86.00 (12.00)	86.00 (12.00)	86.00 (12.00)	88.00 (12.00)	87.00 (12.00)	0.006
Total calcium (mg/dL)	9.40 (0.50)	9.40 (0.50)	9.40 (0.50)	9.40 (0.50)	9.40 (0.50)	0.363
Creatinine (mg/dL)	0.80 (0.20)	0.80 (0.40)	0.80 (0.30)	0.80 (0.30)	0.80 (0.30)	0.169
Categorical variables ^b^						
Male	289 (46.2)	229 (44.5)	242 (47.6)	296 (45.7)	1056 (46.0)	0.784
Ethnicity						<0.001
Mexican American	200 (31.9)	151 (29.3)	126 (24.8)	128 (19.8)	605 (26.3)	
Non-Hispanic white	310 (49.5)	235 (45.6)	238 (46.9)	283 (43.7)	1066 (46.4)	
Non-Hispanic Black	74 (11.8)	91 (17.7)	95 (18.7)	178 (27.5)	438 (19.1)	
Other Hispanic	18 (2.9)	20 (3.9)	27 (5.3)	42 (6.5)	107 (4.7)	
Arthritis	38 (6.1)	39 (7.6)	41 (8.1)	85 (13.1)	203 (8.8)	<0.001
Congestive heart failure	2 (0.3)	1 (0.2)	1 (0.2)	8 (1.2)	12 (0.5)	0.075
Coronary heart disease	3 (0.5)	2 (0.4)	1 (0.2)	5 (0.8)	11 (0.5)	0.573
Angina/Angina pectoris	3 (0.5)	2 (0.4)	4 (0.8)	10 (1.5)	19 (0.8)	0.397
Heart attack Stroke	2 (0.3) 2 (0.3)	1 (0.2) 3 (0.6)	2 (0.4) 2 (0.4)	8 (1.2) 8 (1.2)	13 (0.6) 15 (0.7)	0.128 0.271
Smoke Moderate to vigorous recreational activity	278 (44.4) 363 (58.0)	206(40.0) 275 (53.4)	230 (45.3) 297 (58.5)	306 (47.2) 350 (54.0)	1020 (44.4) 1285 (55.9)	0.131 0.195

^a^ Values were expressed as median (interquartile range)

^b^ Values in the categorical variables were expressed as number (%)

* indicates measles quartiles (Q1, Q2, Q3) with different letters were significantly different from Q4 measles quartile (p < 0.05, ANOVA).

### Measurement of measles antibody

Serum samples from participants who were 6 to 49 years old during 2001–2004 were analyzed with commercially available indirect enzyme-linked immunosorbent IgG assays (IgG ELISA II; Wampole Laboratories) for immunoglobulin G (IgG) antibodies against measles virus. The serostatus of measles IgG antibodies were categorized by index value or optical density ratio (ODR) in the following manner: ODR of ≤ 0.90 as seronegative; ODR of 0.91 to 1.09 as indeterminate; and ODR of ≥ 1.10 as seropositive.[15]

### Determination of vitamin D level

Measurement of serum concentrations of 25(OH)D, including 25(OH)D_3_ and 25(OH)D_2_, was performed in the NHANES participants.[13] For NHANES 1988–1994 and 2001–2006, the serological level of 25(OH)D was measured with a DiaSorin RIA kit (Stillwater MN). In July 2009 the National Institute of Standards and Technology (NIST) used the isotope dilution tandem mass spectrometry (LC-MS/MS) method to set a qualified value of 25(OH)D as the assay standard. The CDC, out of concern over bias and imprecision, developed regression equations to convert the 25(OH)D data from NHANES 2001–2006 in accordance with the aforementioned LC-MS/MS method. The 25(OH)D data from NHANES 2001–2006 were adjusted due to the assay drift and revisions in the reagent and calibration lot.

### Covariates measurement

Several variables concerning personal lifestyle were collected through self-reporting by the participants; the variables included recreational activity, status of tobacco consumption, and physician-diagnosed past medical status, which included arthritis, congestive heart failure, coronary artery disease, angina/angina pectoris, heart attack, and stroke. Moderate to vigorous movement that resulted in an elevation of breathing frequency or heart rates, such as running, swimming or bicycling for more than ten minutes continuously, was defined as recreational activity in our study.

Total serum cholesterol was calculated enzymatically using a Hitachi 704 automatic Analyzer serviced by Roche Diagnostics, Indianapolis, IN. The fasting serum glucose level was determined using a glucose oxidase method with the Roche Gobas Mira biochemistry analyzer. The handling and processing of other biomarkers, such as alanine aminotransferase (ALT), total calcium and creatinine, in the NHANES have been described in other literature.[16] The above practices were executed according to the certified paradigms of CDC.

### Statistical methods

SPSS (Statistical Package for the Social Scientists) was used for data analysis (Version 18.0 for Windows, SPSS Inc., Chicago, IL, USA). Based on the result of Kolmogorov-Smirnov test (p<0.001), serum 25(OH)D and other covariates of our study population were not normal distributed. Therefore, log transformation was performed to normalize the distributions of the serum 25(OH)D. Continuous variables were presented as the medians and interquartile range (IQR), and the Kruskal-Wallis test was used for comparing categorical variables. We categorized the measles antibody titers into quartiles to investigate quartile-based analysis using the highest quartile as the reference group. Multivariate linear regression was used to explore the relationship between vitamin D and the measles antibody titers. Three extended-model approaches with covariate adjustment were utilized. Model 1 was unadjusted, and Model 2 was adjusted for age, sex, and race/ethnicity. Model 3 was comprised of Model 2 + ALT, total cholesterol, glucose, total calcium, and creatinine. Model 3 plus past histories of arthritis, congestive heart failure, coronary heart disease, angina/angina pectoris, heart attack, stroke, smoking status, and moderate to vigorous recreational activity were adjusted in Model 4. All statistical significance was defined as a two-sided P value of <0.05. The R-squared values were listed in Tables 2 and 3 to test whether the multiple regression model is appropriate for our data.

**Table 2:** Association between the level of vitamin D and the level of measles antibody titer.

Models ^a^	ß ^b^ (95% CI)	P value ^c^R^2^
Model 1	-0.008 (-0.010, -0.005)	<0.001 0.001
Model 2	-0.006 (-0.009, -0.004)	<0.001 0.021
Model 3	-0.006 (-0.009, -0.004)	<0.001 0.065
Model 4	-0.006 (-0.009, -0.004)	<0.001 0.070

CI, confidence interval.

^a^ Adjusted covariates: Model 1 = without adjustment. Model 2 = Model 1+ (age, gender, race/ethnicity). Model 3 = Model 2+ (ALT, total cholesterol, glucose, total calcium, creatinine). Model 4 = Model 3+ (history of arthritis, congestive heart failure, coronary heart disease, angina/angina pectoris, heart attack, stroke, smoking, moderate to vigorous recreational activity)

^b^ ß coefficients were interpreted as change of Vitamin D for each unit increase in measles antibody titer.

^c^ Nagelkerke R squared

**Table 3:** Association between vitamin D and measles antibody titer quartiles.

Models ^a^	measles antibody titer quartiles	ß ^b^ (95% CI)	p value	p for trend	^c^R^2^
Model 1	Q3 v.s. Q1 Q2 v.s. Q1	-0.035 (-0.084~0.013)	0.151	<0.001	0.015
	Q3 v.s. Q1	-0.045 (-0.093~0.003)	0.069		
	Q4 v.s. Q1	-0.131 (-0.176~-0.085)	<0.001		
Model 2	Q2 v.s. Q1	-0.030 (-0.078~0.018)	0.226	<0.001	0.029
	Q3 v.s. Q1	-0.034 (-0.083~0.015)	0.176		
	Q4 v.s. Q1	-0.110 (-0.159~-0.061)	<0.001		
Model 3	Q2 v.s. Q1	-0.026 (-0.073~0.021)	0.277	<0.001	0.073
	Q3 v.s. Q1	-0.034 (-0.083~0.014)	0.161		
	Q4 v.s. Q1	-0.109 (-0.157~-0.060)	<0.001		
Model 4	Q2 v.s. Q1	-0.026 (-0.073~0.021)	0.277	<0.001	0.078
	Q3 v.s. Q1	-0.035 (-0.083~0.013)	0.153		
	Q4 v.s. Q1	-0.111 (-0.159~-0.063)	<0.001		

CI, confidence interval.

^a^ Adjusted covariates: Model 1 = age, gender, race/ethnicity. Model 2 = Model 1+ (age, gender, race/ethnicity). Model 3 = Model 2+ (ALT, total cholesterol, glucose, total calcium, creatinine). Model 4 = Model 3+ (history of arthritis, congestive heart failure, coronary heart disease, angina/angina pectoris, heart attack, stroke, smoking, moderate to vigorous recreational activity)

^b^ ß coefficients can be interpreted as differences in Vitamin D comparing subjects in the upper three quartiles to those in the lowest quartiles.

^c^ Nagelkerke R squared

## Results

### Characteristics of the study population

The study group consisted of 5,681 participants; 45.5% were male and the median age of subjects was 34.00 ± 16.00 years old. The demographic characteristics of all subjects classified by quartiles of serum measles antibody titer are presented in Table 1. Older age, lower serum vitamin D levels, higher blood glucose, and higher rates of arthritis were prominent in the highest quartile of the measles antibody titer compared with all the lower quartiles of measles antibody titer.

### Association between measles antibody titer and vitamin D

The measles antibody titer showed significant correlation with the serum concentration of vitamin D. Participants in the highest quartile of measles antibody titer had significantly lower serological levels of 25-hydroxyvitamin D [25(OH)D] than those in the lower quartiles (53.90 vs. 58.70 nmol/L, a decrease of 8.18%) (p < 0.001). The results of linear regression analyses are listed in Table 2. The ß coefficient of 25(OH)D was -0.006 (95% confidence interval, -0.009 ~ -0.004, p<0.001) after adjustment for age, gender, and race/ethnicity. The statistical significance in our study remained when additional covariates were added (ß coefficient = -0.006, p<0.001). Table 3 shows the outcome of multiple linear regression analyses of measles antibody titer quartiles, which demonstrates the negative associations between the serum concentrations of vitamin D and the measles antibody titers. Participants in the higher quartiles of measles antibody titer had significantly lower serum concentrations of vitamin D (p for trend<0.001). Table 4 shows the ethnicity-specific association between the levels of vitamin D and the measles antibody titer in seropositive subjects. (5,480 participants in our study population were seropositive for measles IgG.) The negative association of serum vitamin D levels and measles antibody titers only remained statistically significant (p< 0.05) in Model 1 (without adjustment of age, gender, ethnicity, or other covariates) of non-Hispanic black populations.

**Table 4:** Ethnicity difference in association between level of vitamin D and measles antibody titer in seropositive^b^ subjects.

	Mexican American			Other Hispanic			Non-Hispanic White			Non-Hispanic Black		
Models^a^	β^c^ (95% CI)	P value	R^2^	β^c^ (95% CI)	P value	R^2^	β^c^ (95% CI)	P value	R^2^	β^c^ (95% CI)	P value	R^2^
Model 1	-0.003(-0.008, 0.001)	0.132	0.004	<0.001(-0.011, 0.010)	0.962	<0.001	-0.001(-0.004, 0.002)	0.358	0.001	-0.005(-0.010, <0.001)	0.038	0.010
Model 2	-0.001(-0.006, 0.004)	0.661	0.038	<0.001 (-0.011, 0.012)	0.821	0.004	<0.001 (-0.003, 0.003)	0.979	0.007	-0.005(-0.010, <0.001)	0.066	0.038
Model 3	-0.001(-0.006, 0.003)	0.480	0.104	-0.003(-0.013, 0.008)	0.598	0.221	0.001(-0.003, 0.004)	0.713	0.048	-0.004(-0.009, 0.001)	0.095	0.093
Model 4	-0.002(-0.007, 0.003)	0.393	0.143	-0.001(-0.012, 0.010)	0.859	0.259	0.001(-0.002, 0.004)	0.681	0.066	-0.005(-0.010, 0.001)	0.083	0.114

^a^ Adjusted covariates: Model 1 = without adjustment. Model 2 = Model 1+ age, gender. Model 3 = Model 2 + ALT, total cholesterol, triglycerides, fasting glucose, calcium, creatinine. Model 4 = Model 3+ history of arthritis, congestive heart failure, coronary heart disease, angina/angina pectoris, heart attack, stroke, smoking, moderate to vigorous recreational activity

^b^ Seropositive was defined as optical density ratio (ODR) ≥ 1.10

^c^β coefficient can be interpreted as differences in the change of vitamin D for each unit increase in measles antibody titer.

## Discussion

In this study based on the general US population, a significant negative association was demonstrated between serological level of 25(OH)D and measles antibody titer in the higher quartiles in all models. We suggested that people with higher measles antibody titers tend to have lower serum concentrations of 25(OH)D. As far as we knew, we were the first to address the negative relationship between plasma vitamin D levels and measles antibody titers.

A similar inverse relationship has also been reported between antibody titers against the rubella virus and the season of vaccination.[17] Linder et al. found that children vaccinated in the summer that had higher levels of vitamin D stimulating ultraviolet radiation contained significantly lower geometric mean titers than those who received the vaccination in the winter. A recent study by Zimmerman et al. also demonstrated that lower vitamin D levels were associated with significantly higher antibody titers after three doses of human papillomavirus vaccine.[18] The aforementioned studies and the findings of our present study prompted us to speculate that vitamin D may play a regulatory role in immune response or, more specifically, attenuating the immune reaction induced by viruses, such as measles, rubella, and human papillomavirus. Controversial results of vitamin D on vaccine immunogenicity, however, have also been reported. In one randomized controlled study, serum hemagglutinin inhibition titers were not enhanced in vaccine recipients with simultaneous intramuscular 1,25(OH)D supplement adjacent to the vaccine injection site compared to the placebo group.[19] Another placebo-controlled trial by Moe et al also found no effect of paricalcitol on hepatitis B booster vaccine in hemodialysis patients.[20] Lastly, a recent study by Nicola et al showed that vitamin D level may have no influence on the immune response of trivalent influenza vaccine. In their study, no difference of seroconversion or seroprotection rates was noted between children with persistent low serum vitamin D and those normalized after supplementation.[21]

To date, the mechanism of how vitamin D levels affect the outcome of measles vaccine response remains unclear. A study by Ovsyannikova et al. enrolled 745 healthy school children who had documentation of having two doses of measles, mumps, and rubella (MMR) vaccines and found that specific allelic variants in the vitamin D receptor (VDR) and retinoid X receptor alpha (RXRA) encoding gene were associated with higher measles-virus induced cytokine response.[12] However, the vitamin D levels were not assessed in the study. In our study, we demonstrate the significant inverse relationship between serological levels of 25(OH)D and measles antibody titers.

The activation of an immune response, either by natural infection or vaccination, implicates a delicate interplay of innate and adaptive immune systems;[22] vitamin D can affect both these arms of the immune system. 25(OH)D has been shown to activate the intracrine activity in antigen-stimulated dendritic cells which can then inhibit their maturation and consequently impede downstream T-cell and B-cell proliferation.[23–25] Additionally, 1,25 (OH)D was found to have a potent and direct effect on the B-cells response which hampers B-cell proliferation, generation of class-switched memory B cells, differentiation of plasma cells, and production of immunoglobulins. 25(OH)D exhibits similar properties but at relatively higher concentrations compared with 1,25(OH)D.[26] Such evidence suggests that insufficient vitamin D levels may lead to a more zealous immune response. This is not only consistent with previous studies but also supportive of the inverse relationship between vitamin D levels and measles antibody titers that was observed in our study.

Although several viral infection have been associated with human type 1 diabetes mellitus[27], no solid evidence has indicated that currently used vaccine may induce diabetes in human. In one observational study by Ramondetti, there was no statistical significance between measles infection and type 1 diabetes.[28] The difference of blood glucose level between quartiles in our study, although statistically significant, warrants future study to clarify their correlation. The association between measles and rheumatoid arthritis has been confirmed by previous epidemiological studies.[29, 30] A recent network analysis by Liu et al further explained the association between measles and rheumatoid arthritis by their shared genetic background.[31]

Vitamin D deficiency has been regarded as being pandemic throughout the world.[32] Growing evidences have demonstrated the impact of vitamin D on various constitutional conditions, including autoimmune diseases, cardiovascular diseases, and cancer.[2] Owing to the absence of data regarding the association between measles antibody titer and seral vitamin level, we categorized measles antibody titer into quartiles and revealed a dose-dependent manner of vitamin D level. The fact that the difference of vitamin D levels in the highest and the lowest quartile was small and were both categorized as insufficient was not surprising since participants in NHANES were representative of U.S. general population. Concerning a small difference (53.90 vs. 58.70 nmol/L, a decrease of 8.18%) of vitamin D level in our study has been shown to correlate with significant difference of measles antibody titer, an extrapolation that a greater inverse relationship to measles antibody titer between vitamin D-sufficient (>75 nmol/L) and -deficient (<50 nmol/L) groups would be expected.[33]

There are several limitations in our study that should be considered. First, although the R^2^ value (R^2^ = 0.070) in our study is relatively low, the following reasons are taken into consideration. Sunlight exposure and dietary supplement from several kinds of foods, such as cod liver oil and oily fish, are the main sources of vitamin D for human. [34] The serum concentration of vitamin D, however, is also influenced by multiple factors including age, ethnicity, obesity, medication, various diseases, sunlight exposure, and dietary supplement.[35] The result of our present study may be interpreted that measles antibody is one of these factors that influences the serum concentration of vitamin D which cannot be overlooked. Second, the causal relationship between 25(OH)D levels and the measles antibody titers could not be established since our study was a cross-sectional study. The data was only collected at one time without long-term follow up and the intervention effect could not be elucidated by control group which warrant future randomized placebo-controlled trials. Third, the vaccination status and history of the participants in our study were unknown. The measles antibody titer in vaccinated participants could be much lower than those with natural infection.[36] Lastly, the seral concentration of 25(OH)D could have seasonal and diurnal changes.[37, 38] However, the data from NHANES spanned from 2001 to 2004, and we could not trace the time point when the participants took their blood test.

In summary, this study addresses the negative association between serum 25(OH)D levels and the measles antibody titers. Since no definite causal relationship exists, more attention should be paid to the adequate daily vitamin D supplementation for known health benefits irrespective of measles antibody levels. Additional prospective research focusing on the causal relationship between vitamin D and measles immunity would be of great interest and value.

## Supporting information

S1 Dataset (RAR)

## References

[1] Haussler MR, Whitfield GK, Kaneko I, Haussler CA, Hsieh D, Hsieh JC, et al Molecular mechanisms of vitamin D action. Calcif Tissue Int. 2013;92(2):77–98. 10.1007/s00223-012-9619-0 . 22782502 10.1007/s00223-012-9619-0

[2] Holick MF. Sunlight and vitamin D for bone health and prevention of autoimmune diseases, cancers, and cardiovascular disease. Am J Clin Nutr. 2004;80(6 Suppl):1678s–88s. 10.1093/ajcn/80.6.1678S . 15585788 10.1093/ajcn/80.6.1678S

[3] Lin Z, Li W. The Roles of Vitamin D and Its Analogs in Inflammatory Diseases. Curr Top Med Chem. 2016;16(11):1242–61. . 26369816 10.2174/1568026615666150915111557

[4] Chocano-Bedoya P, Ronnenberg AG. Vitamin D and tuberculosis. Nutr Rev. 2009;67(5):289–93. 10.1111/j.1753-4887.2009.00195.x . 19386033 10.1111/j.1753-4887.2009.00195.x

[5] Ivanov AP, Dragunsky EM, Chumakov KM. 1,25-dihydroxyvitamin d3 enhances systemic and mucosal immune responses to inactivated poliovirus vaccine in mice. J Infect Dis. 2006;193(4):598–600. 10.1086/499970 . 16425140 10.1086/499970

[6] Enioutina EY, Visic D, McGee ZA, Daynes RA. The induction of systemic and mucosal immune responses following the subcutaneous immunization of mature adult mice: characterization of the antibodies in mucosal secretions of animals immunized with antigen formulations containing a vitamin D3 adjuvant. Vaccine. 1999;17(23–24):3050–64. . 10462240 10.1016/s0264-410x(99)00147-4

[7] Centers for Disease Control and Prevention (CDC). Epidemiology of measles—United States, 2001–2003. MMWR Morb Mortal Wkly Rep. 2004;53(31):713–6. . 15306756

[8] Papania MJ, Seward JF, Redd SB, Lievano F, Harpaz R, Wharton ME. Epidemiology of measles in the United States, 1997–2001. J Infect Dis. 2004;189 Suppl 1:S61–8. 10.1086/381557 . 15106091 10.1086/381557

[9] Centers for Disease Control and Prevention (CDC). Measles—United States, 2004. MMWR Morb Mortal Wkly Rep. 2005;54(48):1229–31. . 16340938

[10] Katz SL, Hinman AR. Summary and conclusions: measles elimination meeting, 16–17 March 2000. J Infect Dis. 2004;189 Suppl 1:S43–7. 10.1086/377696 . 15106088 10.1086/377696

[11] Zitt E, Sprenger-Mahr H, Knoll F, Neyer U, Lhotta K. Vitamin D deficiency is associated with poor response to active hepatitis B immunisation in patients with chronic kidney disease. Vaccine. 2012;30(5):931–5. 10.1016/j.vaccine.2011.11.086 . 22142584 10.1016/j.vaccine.2011.11.086

[12] Ovsyannikova IG, Haralambieva IH, Vierkant RA, O'Byrne MM, Jacobson RM, Poland GA. Effects of vitamin A and D receptor gene polymorphisms/haplotypes on immune responses to measles vaccine. Pharmacogenet Genomics. 2012;22(1):20–31. 10.1097/FPC.0b013e32834df186 . 22082653 10.1097/FPC.0b013e32834df186 PMC3237827

[13] Yetley EA, Pfeiffer CM, Schleicher RL, Phinney KW, Lacher DA, Christakos S, et al NHANES monitoring of serum 25-hydroxyvitamin D: a roundtable summary. J Nutr. 2010;140(11):2030s–45s. 10.3945/jn.110.121483 . 20881084 10.3945/jn.110.121483 PMC2955879

[14] Al-Khalidi B, Kimball SM, Rotondi MA, Ardern CI. Standardized serum 25-hydroxyvitamin D concentrations are inversely associated with cardiometabolic disease in U.S. adults: a cross-sectional analysis of NHANES, 2001–2010. Nutr J. 2017;16(1):16 10.1186/s12937-017-0237-6 . 28241878 10.1186/s12937-017-0237-6 PMC5329954

[15] McQuillan GM, Kruszon-Moran D, Hyde TB, Forghani B, Bellini W, Dayan GH. Seroprevalence of measles antibody in the US population, 1999–2004. J Infect Dis. 2007;196(10):1459–64. 10.1086/522866 . 18008224 10.1086/522866

[16] Wu CJ, Kao TW, Tsai CK, Chang YW, Peng TC, Yang HF, et al Was the calf circumference associated with serum vitamin D level in obesity and non-obesity adults. Clin Chim Acta. 2018;481:42–8. 10.1016/j.cca.2018.02.028 . 29481775 10.1016/j.cca.2018.02.028

[17] Linder N, Abudi Y, Abdalla W, Badir M, Amitai Y, Samuels J, et al Effect of season of inoculation on immune response to rubella vaccine in children. J Trop Pediatr. 2011;57(4):299–302. 10.1093/tropej/fmp104 . 19889749 10.1093/tropej/fmp104

[18] Zimmerman RK, Lin CJ, Raviotta JM, Nowalk MP. Do vitamin D levels affect antibody titers produced in response to HPV vaccine? Hum Vaccin Immunother. 2015;11(10):2345–9. 10.1080/21645515.2015.1062955 . 26176493 10.1080/21645515.2015.1062955 PMC4635898

[19] Kriesel JD, Spruance J. Calcitriol (1,25-dihydroxy-vitamin D3) coadministered with influenza vaccine does not enhance humoral immunity in human volunteers. Vaccine. 1999;17(15–16):1883–8. . 10217585 10.1016/s0264-410x(98)00476-9

[20] Moe SM, Zekonis M, Harezlak J, Ambrosius WT, Gassensmith CM, Murphy CL, et al A placebo-controlled trial to evaluate immunomodulatory effects of paricalcitol. Am J Kidney Dis. 2001;38(4):792–802. 10.1053/ajkd.2001.27697 . 11576883 10.1053/ajkd.2001.27697

[21] Principi N, Marchisio P, Terranova L, Zampiero A, Baggi E, Daleno C, et al Impact of vitamin D administration on immunogenicity of trivalent inactivated influenza vaccine in previously unvaccinated children. Hum Vaccin Immunother. 2013;9(5):969–74. 10.4161/hv.23540 . 23324599 10.4161/hv.23540 PMC3899163

[22] Lang PO, Govind S, Bokum AT, Kenny N, Matas E, Pitts D, et al Immune senescence and vaccination in the elderly. Curr Top Med Chem. 2013;13(20):2541–50. . 24066892 10.2174/15680266113136660181

[23] Hewison M, Freeman L, Hughes SV, Evans KN, Bland R, Eliopoulos AG, et al Differential regulation of vitamin D receptor and its ligand in human monocyte-derived dendritic cells. J Immunol. 2003;170(11):5382–90. . 12759412 10.4049/jimmunol.170.11.5382

[24] Alroy I, Towers TL, Freedman LP. Transcriptional repression of the interleukin-2 gene by vitamin D3: direct inhibition of NFATp/AP-1 complex formation by a nuclear hormone receptor. Mol Cell Biol. 1995;15(10):5789–99. . 7565732 10.1128/mcb.15.10.5789 PMC230831

[25] Mahon BD, Wittke A, Weaver V, Cantorna MT. The targets of vitamin D depend on the differentiation and activation status of CD4 positive T cells. J Cell Biochem. 2003;89(5):922–32. 10.1002/jcb.10580 . 12874827 10.1002/jcb.10580

[26] Chen S, Sims GP, Chen XX, Gu YY, Chen S, Lipsky PE. Modulatory effects of 1,25-dihydroxyvitamin D3 on human B cell differentiation. J Immunol. 2007;179(3):1634–47. . 17641030 10.4049/jimmunol.179.3.1634

[27] van der Werf N, Kroese FG, Rozing J, Hillebrands JL. Viral infections as potential triggers of type 1 diabetes. Diabetes Metab Res Rev. 2007;23(3):169–83. 10.1002/dmrr.695 . 17103489 10.1002/dmrr.695

[28] Ramondetti F, Sacco S, Comelli M, Bruno G, Falorni A, Iannilli A, et al Type 1 diabetes and measles, mumps and rubella childhood infections within the Italian Insulin-dependent Diabetes Registry. Diabet Med. 2012;29(6):761–6. 10.1111/j.1464-5491.2011.03529.x . 22133003 10.1111/j.1464-5491.2011.03529.x

[29] Rosenau BJ, Schur PH. Association of measles virus with rheumatoid arthritis. J Rheumatol. 2009;36(5):893–7. 10.3899/jrheum.080856 . 19435971 10.3899/jrheum.080856

[30] Heijstek MW, van Gageldonk PG, Berbers GA, Wulffraat NM. Differences in persistence of measles, mumps, rubella, diphtheria and tetanus antibodies between children with rheumatic disease and healthy controls: a retrospective cross-sectional study. Ann Rheum Dis. 2012;71(6):948–54. 10.1136/annrheumdis-2011-200637 . 22172491 10.1136/annrheumdis-2011-200637

[31] Liu G, Jiang Y, Chen X, Zhang R, Ma G, Feng R, et al Measles contributes to rheumatoid arthritis: evidence from pathway and network analyses of genome-wide association studies. PLoS One. 2013;8(10):e75951 10.1371/journal.pone.0075951 . 24204584 10.1371/journal.pone.0075951 PMC3799991

[32] Cashman KD, Dowling KG, Skrabakova Z, Gonzalez-Gross M, Valtuena J, De Henauw S, et al Vitamin D deficiency in Europe: pandemic? Am J Clin Nutr. 2016;103(4):1033–44. 10.3945/ajcn.115.120873 . 26864360 10.3945/ajcn.115.120873 PMC5527850

[33] Holick MF, Binkley NC, Bischoff-Ferrari HA, Gordon CM, Hanley DA, Heaney RP, et al Evaluation, treatment, and prevention of vitamin D deficiency: an Endocrine Society clinical practice guideline. J Clin Endocrinol Metab. 2011;96(7):1911–30. 10.1210/jc.2011-0385 . 21646368 10.1210/jc.2011-0385

[34] Holick MF. Sunlight "D"ilemma: risk of skin cancer or bone disease and muscle weakness. Lancet. 2001;357(9249):4–6. 10.1016/S0140-6736(00)03560-1 . 11197362 10.1016/S0140-6736(00)03560-1

[35] Tsiaras WG, Weinstock MA. Factors influencing vitamin D status. Acta Derm Venereol. 2011;91(2):115–24. 10.2340/00015555-0980 . 21384086 10.2340/00015555-0980

[36] Itoh M, Okuno Y, Hotta H. Comparative analysis of titers of antibody against measles virus in sera of vaccinated and naturally infected Japanese individuals of different age groups. J Clin Microbiol. 2002;40(5):1733–8. 10.1128/JCM.40.5.1733-1738.2002 . 11980952 10.1128/JCM.40.5.1733-1738.2002 PMC130661

[37] Holick MF. McCollum Award Lecture, 1994: vitamin D—new horizons for the 21st century. Am J Clin Nutr. 1994;60(4):619–30. 10.1093/ajcn/60.4.619 . 8092101 10.1093/ajcn/60.4.619

[38] Webb AR, Kline L, Holick MF. Influence of season and latitude on the cutaneous synthesis of vitamin D3: exposure to winter sunlight in Boston and Edmonton will not promote vitamin D3 synthesis in human skin. J Clin Endocrinol Metab. 1988;67(2):373–8. 10.1210/jcem-67-2-373 2839537 10.1210/jcem-67-2-373

